# Clinical and subclinical endometritis induced alterations in bovine endometrial transcriptome and miRNome profile

**DOI:** 10.1186/s12864-016-2513-9

**Published:** 2016-03-10

**Authors:** Dessie Salilew-Wondim, Sally Ibrahim, Samuel Gebremedhn, Dawit Tesfaye, Maike Heppelmann, Heinrich Bollwein, Christiane Pfarrer, Ernst Tholen, Christiane Neuhoff, Karl Schellander, Michael Hoelker

**Affiliations:** Department of Animal Breeding and Husbandry, Institute of Animal Science, Endenicher Allee 15, 53115 Bonn, Germany; Clinic for Cattle, University of Veterinary Medicine, 30173 Hannover, Germany; University of Veterinary Medicine, 30173 Hannover, Germany; Clinic of Reproductive Medicine, Vetsuisse Faculty, University of Zurich, 8057 Zurich, Switzerland

**Keywords:** Endometritis, Bovine, Transcriptome, miRNome, Inflammation

## Abstract

**Background:**

Clinical and subclinical endometritis are known to affect the fertility of dairy cows by inducing uterine inflammation. We hypothesized that clinical or subclinical endometritis could affect the fertility of cows by disturbing the molecular milieu of the uterine environment. Here we aimed to investigate the endometrial molecular signatures and pathways affected by clinical and subclinical endometritis. For this, Holstein Frisian cows at 42–60 days postpartum were classified as healthy (HE), subclinical endometritis (SE) or clinical endometritis (CE) based on veterinary clinical examination of the animals and histological evaluation the corresponding endometrial biopsies. Endometrial transcriptome and miRNome profile changes and associated molecular pathways induced by subclinical or clinical endometritis were then investigated using GeneChip® Bovine Genome Array and Exiqon microRNA PCR Human Panel arrays, respectively. The results were further validated in vitro using endometrial stromal and epithelial cells challenged with subclinical and clinical doses of lipopolysaccharide (LPS).

**Result:**

Transcriptome profile analysis revealed altered expression level of 203 genes in CE compared to HE animals. Of these, 92 genes including *PTHLH, INHBA, DAPL1* and *SERPINA1* were significantly upregulated, whereas the expression level of 111 genes including *MAOB*, *CXCR4*, *HSD11B* and*, BOLA*, were significantly downregulated in CE compared to the HE animal group. However, in SE group, the expression patterns of only 28 genes were found to be significantly altered, of which 26 genes including *PTHLH, INHBA, DAPL1, MAOB*, *CXCR4* and *TGIF1* were common to the CE group. Gene annotation analysis indicated the immune system processes; G-protein coupled receptor signaling pathway and chemotaxis to be among the affected functions in endometritis animal groups. In addition, miRNA expression analysis indicated the dysregulation of 35 miRNAs including miR-608, miR-526b* and miR-1265 in CE animals and 102 miRNAs including let-7 family (let-7a, let-7c, let-7d, let-7d*, let-7e, let-7f, let-7i) in SE animals. Interestingly, 14 miRNAs including let-7e, miR-92b, miR-337-3p, let-7f and miR-145 were affected in both SE and CE animal groups. Further in vitro analysis of selected differentially expressed genes and miRNAs in endometrial stroma and epithelial cells challenged with SE and CE doses of LPS showed similar results to that of the array data generated using samples collected from SE and CE animals.

**Conclusion:**

The results of this study unraveled endometrial transcriptome and miRNome profile alterations in cows affected by subclinical or clinical endometritis which may have a significant effect on the uterine homeostasis and uterine receptivity.

**Electronic supplementary material:**

The online version of this article (doi:10.1186/s12864-016-2513-9) contains supplementary material, which is available to authorized users.

## Background

During the early lactation period, the majority of high producing dairy cows usually enter in a state of negative energy balance due to reduced voluntary feed intake [1, 2]. This phenomenon in turn compromises the host innate and acquired defense mechanisms and the cows then become susceptible to various uterine disorders [3, 4] and bacterial infections that could cause subclinical and clinical endometritis [3, 5–8]. Indeed, during bacterial infection of the uterus, the immune cells along with the endometrial epithelial and storma1cells generate the immune response of the uterus against the invading pathogens [9]. The innate pathogen defense mechanism is believed to be involved in complicated molecular mechanisms, while contracting with foreign bodies. Therefore, unravelling the endometrial molecular pathways and functional alteration that could be triggered during uterine infection may help to identify biomarkers associated with clinical and/or subclinical endometritis. So far, only very few attempts have been conducted to identify molecular signatures associated with subclinical or clinical endometritis in cattle. For instance, increased levels of interleukin 6 (IL6), interleukin 8 (IL8), tumour necrosis factor alpha (*TNFA*) and β-actin expression levels have been reported in cows with endometrial proportion of polymorphonuclear neutrophils (PMNs) higher than 18 % [10]. Similarly, increased levels of *IL1A* and *IL1RN* in cows affected with subclinical or clinical endometritis have been reported [11]. Apart from the cytokines, understanding the global transcriptome profile changes occurring in the endometrium is essential to understand the impact of endometritis on uterine gene expression landscape. On this regard, previously, we have detected slight endometrial gene expression changes in dairy cows classified as sub clinically sick with endometritis based on endometrial PMN proportion of cytobrush samples [12]. In that study, however, the classification scheme used to categorize cows as sub clinically sick or healthy seems to be very weak to discriminate molecular alterations that could occur in animals affected by endometritis. Thus, here we thought that using a combination of veterinary clinical examination of the animals along with the histological analysis of the corresponding endometrial biopsy could be a better option to investigate the molecular and biochemical changes that could occur in the endometrium due to subclinical or clinical endometritis. Therefore, the present study aimed to investigate the endometrial transcriptome profile changes (mRNA and miRNA expression levels) and associated molecular pathways in dairy cows classified as subclinical or clinical endometritis based on clinical diagnosis and histological evaluation of the endometrial biopsies.

## Results

### Incidence of subclinical or clinical endometritis

To comprehend the prevalence of subclinical and clinical endometritis, first clinical diagnosis was performed in each of the 45 cows by an experienced veterinarian and the corresponding endometrial biopsies were subjected to histological assessment. Due to insufficient quality of their biopsies, seven cows were excluded from further analysis. Out of the remaining 38 cows, 71.1 % were classified as healthy based on the results of clinical diagnosis and histological evaluation of the corresponding endometrial biopsies. In contrast, 6 out of the 38 (15.8 %) cows were found to be clinically healthy whereas the histological examination of their corresponding endometrial biopsies indicated the presence of acute and chronic forms of endometritis (presence lymphocytes or/and granulocytes). On the other hand, both the clinical diagnosis and histological assessment on their biopsy also indicated that 13.2 % of the cows were affected by endometritis whereas 5.3 % the cows diagnosed as clinically sick were found to be healthy based on histological investigation of the endometrial biopsies. Therefore, those clinically sick cows with the presence of chronic and/or acute endometrial inflammation on the corresponding endometrial biopsies were considered as affected by clinical endometritis (CE). Clinically healthy animals with acute and/or chronic endometritis on their endometrial cytology were considered as subclinical endometritis (SE) group and those animals which didn’t show a sign of sickness during clinical diagnosis and histological evaluation of their endometrial biopsies were considered as healthy (HE) group.

### Endometrial gene expression changes in cows affected by clinical endometritis

**Fig. 1:**
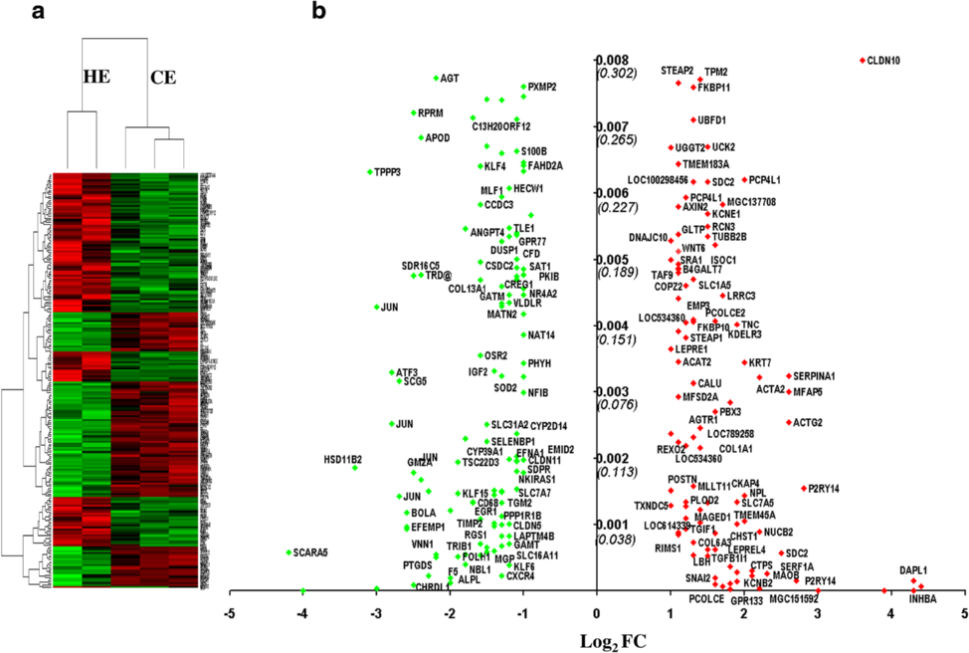
Dysregulated genes in CE compared to HE animals. **a** The expression patterns and the hierarchical clustering of 203 genes within and between the biological samples of CE and HE animal groups. The red and green colors indicate the up and downregulated gene expression patterns, respectively. **b** The fold change and *p* value of 111 downregulated (*green dots*) and 92 upregulated genes (*red dots*) in CE compared to HE animals. The names of some genes are indicated and some others are omitted to enhance visibility. Log_2_FC on the X axis indicates the fold change difference in log _2_ scale. Positive log_2_FC values represent the upregulation of genes, while negative log_2_FC values indicate downregulation of genes in CE compared to the HE animal group. The Y axis indicates the *p* value (*bold faced*) and FDR (*italics in bracket*)

To investigate the effect of clinical endometritis on the endometrial gene expression, the total RNAs isolated from endometrial biopsies of CE and HE animal groups were amplified, biotin labelled and hybridized to the GeneChip® Bovine Genome Array (Affymetrix, CA, USA). Three hybridizations were performed for each of the CE or HE group and the normalized signals intensities of the CE animal group were compared to the normalized signals intensities of the HE group. During analysis, one of the arrays in the HE group didn’t pass the quality control parameters and thus it was removed from the analysis. The gene expression profile showed that the expression levels of 203 gene transcripts were dysregulated (absolute fold change ≥ 2, *p* ≤ 0.008 and false discovery rate (FDR) ≤ 0.30) in CE compared to HE animals (Fig. 1a, Additional file 1: Table S1). Of these, the expression levels of 92 genes including *PTHLH, INHBA, DAPL1, CLDN10, P2RY14* and *MAOB* were increased while the mRNA expression levels of 111 genes including *SCARA5, HSD11B2, TPPP3, JUN, ATF3, BOLA* and *PTGDS* were downregulated in the CE animals (Fig. 1b).

### Endometrial gene expression changes in cows affected by subclinical endometritis

**Fig. 2:**
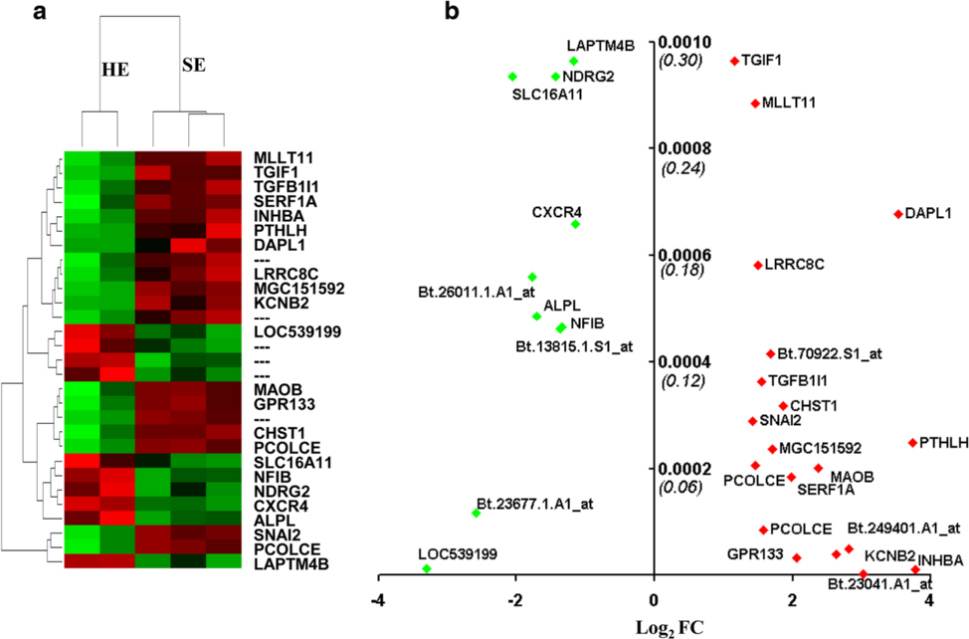
The list of differentially expressed genes between SE and HE animal groups. **a** The expression patterns and the hierarchical clustering of 28 genes within and between the biological samples. The red and green colors indicate up and downregulated gene expression patterns, respectively. **b** The fold change and *p* value of downregulated genes (*green dots*) and the upregulated genes (*red dots*) in SE compared to HE. The names of some genes are indicated and some others are omitted to enhance visibility. The Affymetrix ID is provided for the transcripts which have no official gene symbol. Log_2_FC on the X axis indicates the fold change difference in log_2_ scale. Positive log_2_FC values represent the upregulation while negative log_2_FC values indicate downregulation of genes in SE animals compared to the HE group. The Y axis indicates the *p* value (*bold faced*) and the FDR (*italics in bracket*)

To investigate the effects of subclinical endometritis on the endometrial transcriptome profile of postpartum dairy cows, the endometrial gene expression patterns of the SE animals were compared with the endometrial gene expression patterns of the HE animals using a similar protocol described above for the CE animals. Accordingly, from a total of 8472 genes that exhibited a high signal intensity above the background, 483 gene transcripts were altered by ≥ 2 fold regulation in SE groups compared to HE ones, of which the expression levels of 231 genes were increased while the expression levels of 252 transcripts were reduced in the former compared to the latter group. However, the expression level of only 28 gene transcripts were significantly differentially expressed between SE and HE animals. From these, *PTHLH, INHBA, DAPL1, KCNB2, MAOB* and *GPR133* were the top among the upregulated genes, while *MAMDC2*, *SLC16A1, ALPL, NDRG2, NFIB, LAPTM4B, CXCR4* and *TGIF1* were the top among the suppressed ones in the SE animal group (Fig. 2, Additional file 2: Table S2).

### Genes affected both in subclinical and clinical endometritis animal groups

**Fig. 3:**
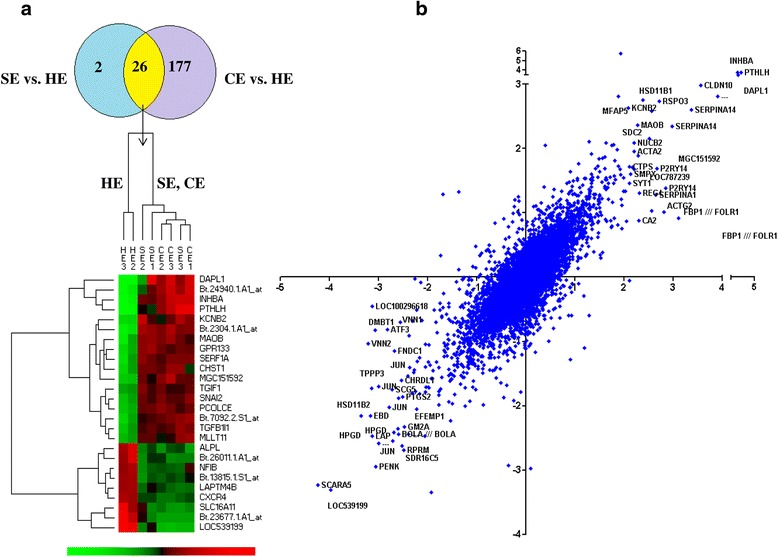
Significantly dysregulated genes both in SE and CE animals compared to HE group. **a** The heatmap illustrating the expression patterns of 26 commonly dysregulated genes in SE and CE animals. The red and green colors in the heatmap indicate high and low expression level of genes, respectively. **b** The scatter plot showing the relative expression patterns of 8472 genes in SE and CE compared to the HE animal group. The relative expression of SE compared to the HE group (Log_2_ fold change) is indicated in the X axis while the relative expression of CE animals compared to the HE group (log_2_ fold change) is plotted in the Y axis. Negative values in the X and Y axis indicated the downregulation of genes while positive values indicated upregulated genes

After identification of differentially expressed genes in CE and SE animals, we merged the two data to identify the common genes that were affected in both animal groups. Interestingly, 26 of the 28 significantly altered genes in SE group were also significantly dysregulated in CE animal group (Fig. 3a). However, the expression levels of 177 significantly altered genes in CE were not significantly affected in the SE animals (Fig. 3a).

Apart from significantly differentially expressed genes, we also looked into the expression trends of all 8472 genes, which exhibited high signal intensity above the background to get a broader overview about the overall gene expression tendencies in SE and CE with reference to the healthy animals. The result of this analysis indicated that the gene expression patterns in SE and CE animals against the HE groups were found to show a similar direction and thus those genes which were downregulated or upregulated in SE were found to be downregulated or upregulated in CE animal group only with a few exceptions (Fig. 3b).

### Functional classification of dysregulated genes

To get insight about the functional alterations that could occur in SE or CE animals due to the dysregulated genes, gene ontology enrichment analysis was performed using the G profiler software. Immune system process, cell adhesion, regulation of neurogenesis, regulation of apoptotic signaling pathway, G-protein coupled receptor signaling pathway and chemotaxis were found to be affected in animals affected by endometritis. Furthermore, except INHBA, all differentially expressed genes which are involved in immune system process were downregulated in cows affected by endometritis (Table 1).

**Table 1:** Functional annotations enriched by the differentially expressed genes

Functional annotations	*P* value	List of genes
Response to stimulus	<0.0001	↑WNT6, ↓VNN1, ↓TSC22D3, ↓TRIB1, ↑TNC, ↓TLE1, ↓TIMP2, ↑TGIF1, ↑TGFB1I1, ↑STEAP2, ↑SRA1, ↓SOD2, ↑SNAI2, ↑PTHLH, ↓PTGDS, ↓PPP1R1B, ↑POSTN, ↓NKIRAS1, ↓NFIB, ↓NBL1,↑MLLT11, ↓KLF6, ↓KLF4, ↑KCNE1, ↓JUN, ↑INHBA, ↓IGF2, ↓GPR77, ↓F5, ↓EGR1, ↓EFNA1, ↑DNAJC10, ↓CXCR4, ↓CD68, ↑AXIN2, ↓ANGPT4, ↓ALPL, ↓ALDH1A3, ↑AGTR1, ↓AGT, ↑ACTG2
Immune system process	0.0467	↓VNN1, ↓TSC22D3, ↓SOD2, ↓S100B, ↓NBL1, ↓MLF1, ↓KLF4, ↓JUN, ↑INHBA, ↓IGF2, ↓GPR77, ↓EGR1, ↓CXCR4, ↓CFD, ↓APOD
Cell adhesion	0.0009	↓VNN1, ↓TGM2, SNAI2, ↑POSTN, KLF4, ↓F5, ↓EMID2, ↓EFNA1, ↑COL1A1, ↓COL13A1, ↓B4GALNT2, ↓APOD, ↓AGT
Response to endogenous stimulus	0.0115	↑TGFB1I1, ↑STEAP2, ↑SNAI2, ↓PPP1R1B, ↓NR4A2, ↑KCNE1, ↓JUN, ↓IGF2, ↓EGR1, ↑COL1A1
Enzyme linked receptor protein signaling pathway	0.0035	↑TGIF1, TGFB1I1, ↓NBL1, ↓JUN, ↑INHBA, ↓IGF2, ↓EGR1, ↓EFNA1, ↓APOD, ↓AGT
Blood vessel development	0.0015	SAT1, KLF4, ↓JUN, ↓EGR1, ↓EFNA1, ↓CXCR4, ↑COL1A1, ↓ANGPT4, ↓AGT, ↑ACTG2
Regulation of neurogenesis	0.0199	↓VLDLR, ↓TIMP2, ↑TGIF1, SDC2, ↓NBL1, ↓EFNA1, ↓CXCR4
Regulation of apoptotic signaling pathway	0.00703	↓VNN1, ↓SOD2, ↑SNAI2, ↑MLLT11, ↑MAGED1, ↑INHBA, ↓AGT
G-protein coupled receptor signaling pathway	0.0212	↓SCG5, ↓RGS1, ↑PTHLH, ↑GPR133, ↑AXIN2, ↑AGTR1, ↓AGT
Chemotaxis	0.0125	↓NFIB, ↓NBL1, ↓MATN2, ↓GPR77, ↓EFNA1, ↓CXCR4, ↑AGTR1

Symbols: ↑ and ↓ indicate the up and downregulation of gene expression, respectively

### Validation of candidate differentially expressed gene using quantitative reverse transcription PCR (qRT-PCR)

The the gene expression differences between SE and HE, CE and HE revealed by the microarray analysis were further confirmed using qRT-PCR. To do this, 13 differenitally expressed genes incldung *P2RY14, INHBA, MAOB, KCNB2, PTHLH, NKIRAS1, TRIB1* and *CXCR4* were randomly selected and the transcript abaundances of these genes in samples derived from SE, CE and HE animal groups were measured using qRT-PCR. Accordingly, the results obtained from qRT-PCR analyses were in agreement with the array results (Table 2). In additon, the microarray and the qRT-PCR results were positively correlated with correlation coffient of 0.8.

**Table 2:** Validation of differentially expressed genes between CE vs. HE or SE vs. HE using qRT-PCR

Gene symbol	Array result		qRT-PCR result		Groups
	Fold change	*P* value	Fold change	*P* value	
*P2RY14*	6.9	0.001	1.96	0.00016	CE vs HE
*INHBA*	19.2	0.000003	5.29	0.0045	
*MAOB*	6.3	0.0002	1.69	0.26	
*KCNB2*	4.2	0.0002	3.43	0.0002	
*MGC151592*	4.4	0.00003	2.29	<0.0001	
*CTPS*	4.2	0.0003	2.03	0.12	
*PTHLH*	21.1	0.00006	9.93	0.0001	
*NKIRAS1*	-2.3	0.0020	-2.29	0.017	
*TRIB1*	-3.1	0.0005	-3.39	0.001	
*CXCR4*	-2.5	0.0002	-2.57	0.007	
*F5*	-4.1	0.00020	-3.4	0.06	
*PTGDS*	-4.9	0.00023	-3.43	0.018	
*CHRDL1*	-5.8	0.00009	-3.85	0.007	
*MAOB*	2.36	0.0002	4.0	0.008	SE vs HE
*KCNB2*	2.62	0.00003	10.5	0.00003	
*MGC151592*	1.71	0.00023	2.13	0.003	

Positive and and negative values indicate up and down regulation genes in SE or CE compared to HE

### The experssion patterns of selected candidate differentially expressed genes in endometrial epithelial and stroma cells challenged with lipopolysacharide (LPS) in vitro

**Fig. 4:**
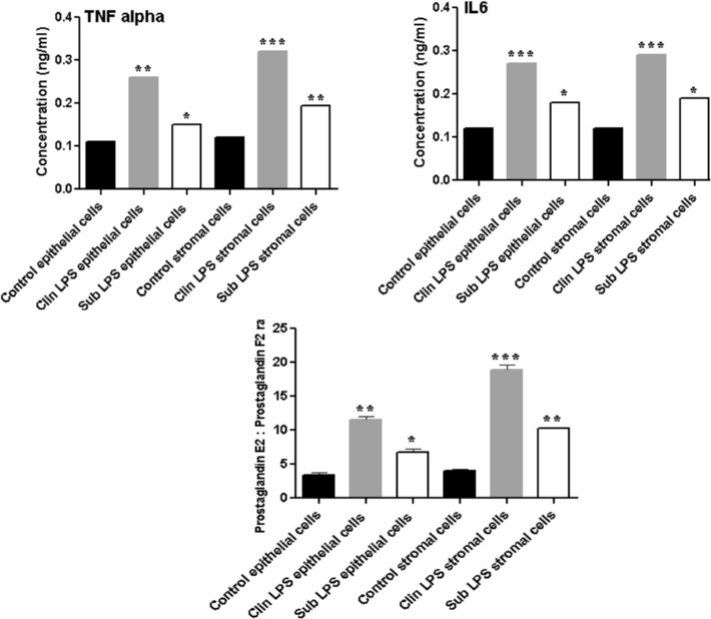
The protein level of TNF alpha & IL6 and PGE2: PGF2aa ratio in the cell culture supernatant measured using ELISA in endometrial cells challenged with clinical and subclinical doses of LPS. Control epithelial and control stroma cells describe unchallenged epithelial and stroma cells, respectively. Clin LPS epithelial cells and clin LPS stroma cells designate endometrial epithelial cells and stroma cells challenged with CE equivalents of LPS, respectively whereas sub LPS epithelial cells and sub LPS stroma cells describe the endometrial epithelial cells and stroma cells challenged with SE equivalents of LPS, respectively. * *P* < 0.05, ** *P* < 0.01, *** *P* < 0.001

**Fig. 5:**
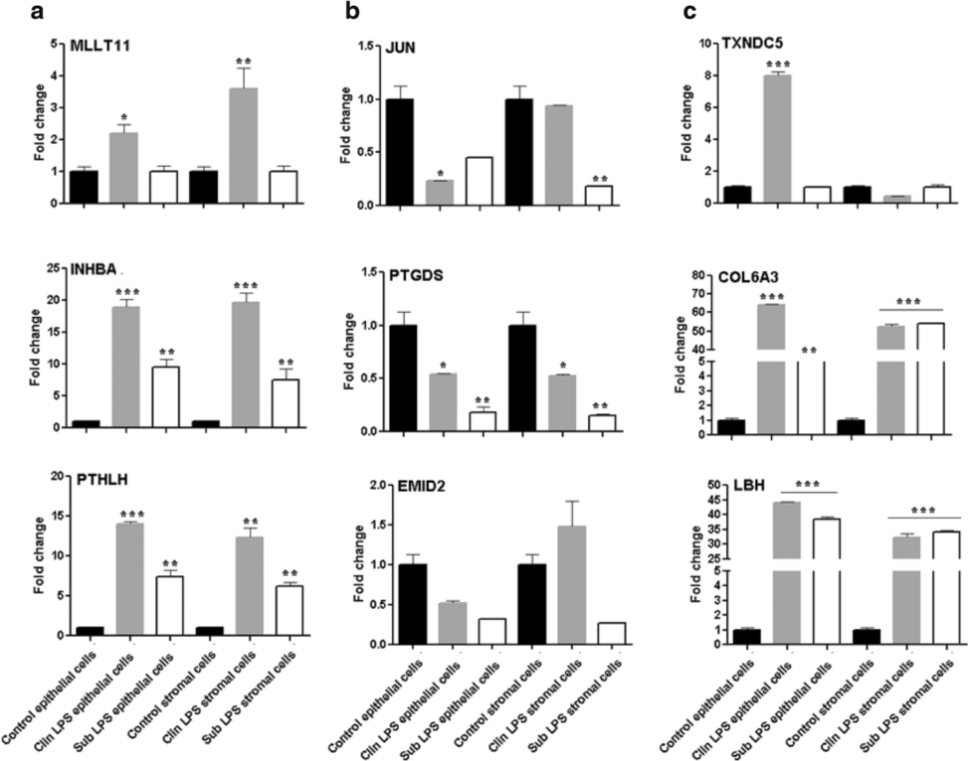
The expression pattern of candidate genes in the endometrial epithelial and stroma cells challenged with CE and SE doses of LPS in vitro for those upregulated both in SE and CE (**a**), downregulated in SE (**b**) or upregulated in CE group (**c**). Control epithelial and control stroma cells describe unchallenged epithelial and stroma cells, respectively. Clin LPS epithelial cells and clin LPS stroma cells designate endometrial epithelial cells and stroma cells challenged with CE equivalents of LPS, respectively whereas sub LPS epithelial cells and sub LPS stroma cells describe the endometrial epithelial cells and stroma cells challenged with SE equivalents of LPS, respectively. * *P* < 0.05, ** *P* < 0.01, *** *P* < 0.001

Transcriptome profile alterations detected in SE or CE animals by the microarray and qRT-PCR analyses were further evaluated in endometrial cells challenged with SE or CE equivalent doses of LPS in vitro. To achieve this, endometrial stromal and epithelial cells collected from healthy non pregnant cows were challenged with clinical (3 μg/ml) or subclinical (0.5 μg/ml) doses of LPS. After the challenge, a significant increase in TNFα and IL-6 protein levels and a higher PGE_2_ to PGF_2α_ ratio were observed in epithelial and stromal spent culture media indicating the effectiveness of LPS challenge to induce inflammation in both cell types (Fig. 4). We then analyzed the expression patterns of nine candidate genes in LPS challenged endometrial epithelial and endometrial cells. The results showed that the expression patterns of three genes (*MLLlT11, INHBA* and *PTHLH*) which were increased in both SE and CE animals were also found to be upregulated in both endometrial epithelial and stroma cells challenged with SE and CE equivalent of LPS (Fig. 5a). Similarly, the expression profiles of three candidate genes (*JUN*, *PTGDS, EMID2)* which exhibited a significant reduction in CE animals were also reduced in endometrial epithelial cells challenged with both SE and CE equivalent doses of LPS (Fig. 5b). In addition, among the three candidate genes (*TXNDC, COL6A3, LBH)* that were significantly increased only in CE animal group, the expression levels of COL6A3 and *LBH* were increased in both cell types challenged by SE and CE equivalents of LPS doses while the expression level of TXNDC gene was increased only in epithelial cells challenged with CE doses of LPS (Fig. 5c).

### MiRNAs detected in bovine endometrium

**Fig. 6:**
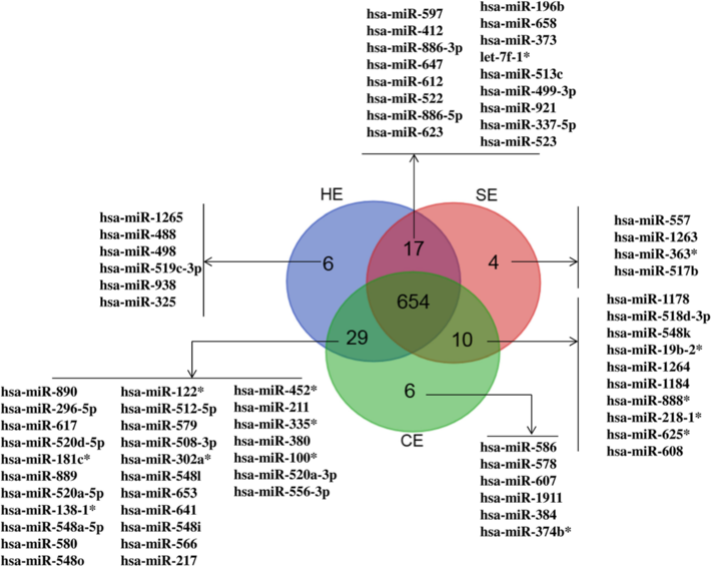
Venn diagram indicating exclusively and commonly expressed miRNAs in SE; CE and HE animal groups

To understand the effects of endometritis on endometrial miRNA expression profile, the total RNA sample used for mRNA expression analysis in CE, SE and HE animals were subjected to miRNA expression analysis using human miRCURY LNA™ Universal RT microRNA PCR array system (Exiqon). The expression patterns of 742 miRNAs in SE or CE animals were compared to the HE ones. For this, only miRNAs detected in less than 36 cycles of PCR amplification were considered as detected miRNAs while those appeared after 36 cycles were considered as undetected ones. Based on these criteria, a total of 706, 685 and 699 miRNAs were detected in HE, SE and CE animal groups, respectively of which 654 miRNAs were detected in all animal groups. Although 93–96 % of the detected miRNAs were common in all groups, some miRNAs detected in HE animals were absent in SE or CE groups and vice-versa (Fig. 6). Among these, miR-938, miR-519c-3p, miR-1265, miR-498 and miR-488 were exclusively detected only in HE animals and 10 miRNAs including miR-608, miR-625*, miR-218-1*, miR-888*, miR-1184 and miR-1264 were detected only in SE and CE animal groups. However, 29 miRNAs such as miR-890*, miR-296-5p, miR-617, miR-181c*, miR-889, miR-520a-5p and miR-641 were absent in SE but these miRNAs were detected in HE and CE animals (Fig. 6).

### Endometrial miRNA expression profile in cows affected by clinical endometritis

**Fig. 7:**
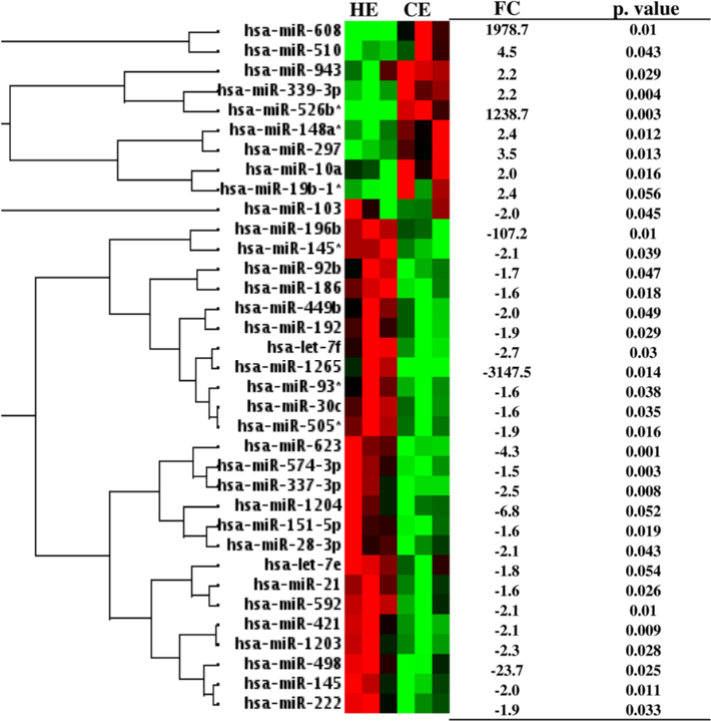
The heatmap describing the expression patterns and hierarchical clustering of differentially expressed miRNAs between CE and HE animals. The red and green colors indicate high and low expression patterns, respectively. Thee biological replicates were used for each animal group. FC = fold change

To investigate the effect of clinical endometritis on the endometrial miRNA expression profile, the miRNA expression levels of CE animals were analyzed with reference to the miRNA expression patterns of HE animals. For this, the miRNA expression data was analyzed using the RT^2^ profiler PCR array data analysis tool, http://pcrdataanalysis.sabiosciences.com/pcr/arrayanalysis.php. The result indicated that the expression levels of 35 miRNAs were significantly altered (absolute fold change *>* 1.5, *p* < 0.05) in CE compared to the HE animals (Fig. 7) of which the expression levels of 7 miRNAs were significantly increased, while the expression levels of 28 miRNAs were reduced in CE group. The absolute fold change of differentially expressed miRNAs ranged between 1.5 and 3147. For instance, miR-608 and miR-526b* were the top among the upregulated miRNAs while miR-1265, miR-196b, miR-498 and miR-1204 were the top among the downregulated miRNAs in CE animal group (Fig. 7). The hierarchical clustering, the expression pattern and the fold changes differences along with the corresponding *p* values of the differentially expressed miRNAs are presented in Fig. 7.

### Endometrial miRNA expression pattern in cows affected by subclinical endometritis

To comprehend the consequence of subclinical endometritis on the endometrial miRNA expression profile, the miRNA expression of SE animal was compared to the HE following the same methodological approach employed for the CE group. Accordingly, the expression level of 102 miRNAs were significantly differentially expressed (absolute fold change *>* 1.5, *p* < 0.05) between the SE and HE animal groups. Of these, the expression levels of 11 miRNAs were significantly increased while the expression levels of 95 miRNAs were reduced in SE animal group (Additional file 3: Figure S1, Additional file 4: Table S3). Moreover, miR-361-5p, miR-1184 and miR-218-1* were the top among the upregulated miRNAs while miR-1265, miR-20b*, miR-520d-5p and miR-506 were the top among the downregulated miRNAs in the SE animals. Interestingly, the expression level of certain miRNA families namely, the let 7 family (let-7a, let-7c, let-7d, let-7d*, let-7e, let-7f, let-7i), miR-181 family (miR-181a, miR-181b), miR-30 family *(*miR-30b*, miR-30c-2*, miR-30e*)*, miR-425 family (miR-425, miR-425*), miR-92 family (miR-92a, miR-92a-1*, miR-92b) and miR-196 family (miR-196a, and miR-196b) were repressed in SE animal group.

### MiRNAs dysregulated in both subclinical and clinical endometritis

**Fig. 8:**
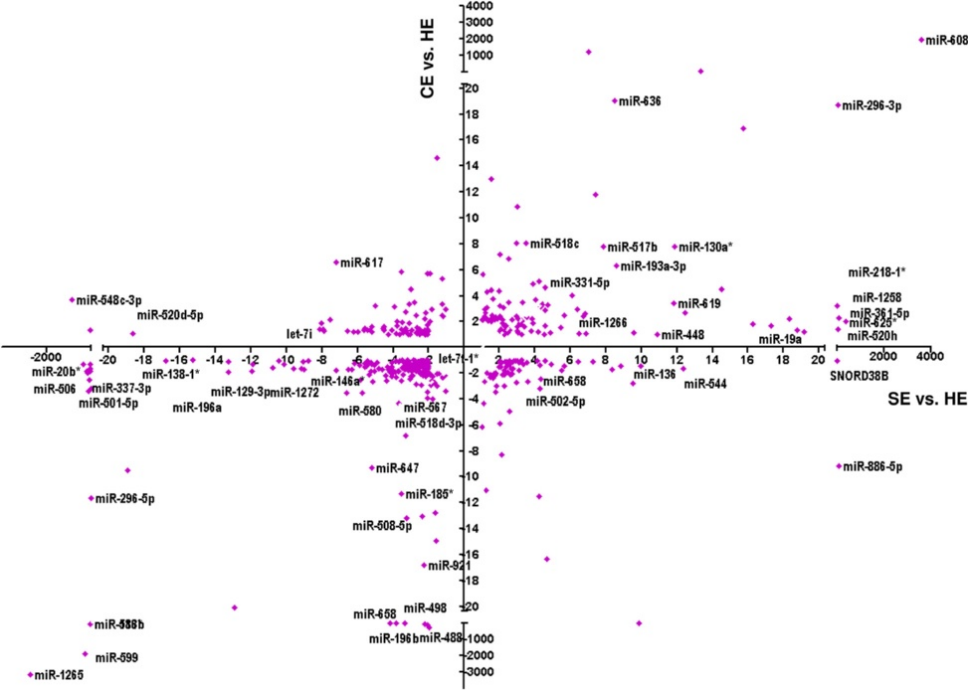
The scatter plot showing the expression patterns of 742 miRNAs in SE and CE compared to the HE animal group. The relative expression of SE compared to HE (Log_2_ fold change) is indicated in the X axis while the relative expression of CE animal groups relative to HE (log_2_ fold change) is plotted in the Y axis. Negative log_2_ fold and positive log_2_ fold change values in the X and Y axis indicated the upregulated and downregulation of miRNAs, respectively. Only the names of some dysregulated miRNAs are indicated to enhance visibility

**Fig. 9:**
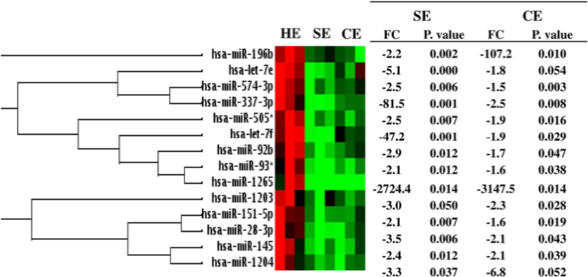
The heatmap indicating the expression patterns of the dysregulated miRNAs both in SE and CE compared to HE animal group. The *red* and *green* colors indicate high and low miRNA expression, respectively. FC = the relative expression in fold changes

Since both SE and CE animals were affected by endometritis, we further extended our analysis to identify miRNAs affected in both animal groups. To perform this, we considered all miRNAs detected in all animal groups. This analysis has revealed that, the expression pattern of miRNAs in SE and CE animal groups tended to show a similar pattern when the expression level of both animal groups were compared to the expression level of the HE animals. From these, the expression levels of 120 miRNAs exhibited ≥ 2 fold regulations in SE and CE animals. Of these, the expression levels 47 and 51 miRNAs were found to be up and downregulated, respectively in both animal groups compared to the HE ones and the expression levels of 32 miRNAs showed opposite trend in SE and CE animals (Fig. 8). Nevertheless, when the criteria were set to absolute fold change *>* 1.5 and *p* < 0.05, only 14 miRNAs were commonly significantly differentially expressed in both SE and CE animals compared to the HE groups (Fig. 9).

### Experssion analysis of candidate miRNAs in endometrial epithelial and stroma cells challenged with lipopolysacharide (LPS) in vitro

**Fig. 10:**
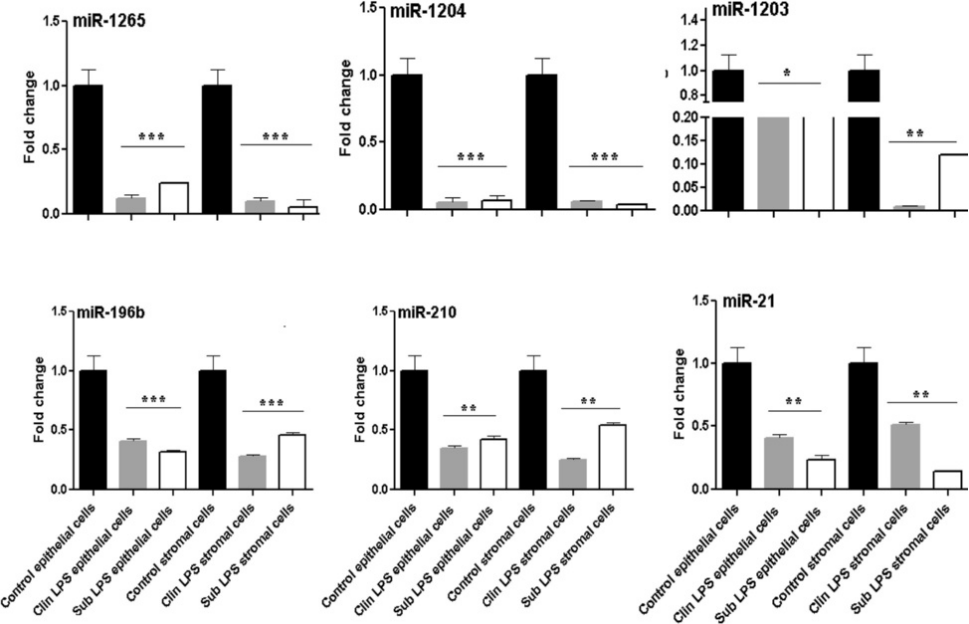
The expression patterns of candidate miRNAs in endometrial epithelial and stroma cells challenged with clinical and subclinical doses of LPS in vitro. Control epithelial and control stroma cells describe unchallenged epithelial and stroma cells, respectively. Clin LPS epithelial cells and clin LPS stroma cells designate endometrial epithelial cells and stroma cells challenged with CE equivalents of LPS, respectively whereas sub LPS epithelial cells and sub LPS stroma cells describe the endometrial epithelial cells and stroma cells challenged with SE equivalents of LPS, respectively. * *P* < 0.05, ** *P* < 0.01, *** *P* < 0.001

Endometrial miRNA expression pattern alteration identified in SE and CE animals using PCR based miRNA platform arrays were further validated in bovine endometrial cells challenged with clinical and subclinical endometritis equivalent doses of LPS in vitro. For this, 6 candidate miRNAs, namely those downregulated both in SE and CE animals (miR-1265, miR-1204, miR-1203 and miR-196b), downregulated only in SE animals (miR-210) or only in CE animals (miR-21) were quantified in LPS challenged epithelial and stromal endometrial cells. The expression levels of these candidate miRNAs were found to be reduced in both epithelial and stromal endometrial cells challenged with clinical and subclinical equivalents of LPS doses (Fig. 10).

### Comparative analysis of differentially expressed miRNAs and differentially expressed genes

After performing the microRNA and mRNA expression profiles from the same sample, the relationship between differentially expressed miRNAs and mRNAs was in silico predicted to understand whether the affected miRNAs in SE and CE animal groups could have a possible association with differentially expressed genes. For this, the corresponding target genes of the differentially expressed miRNAs were predicted using the web-based miRNA- target gene prediction tool (http://c1.accurascience.com/miRecords/). Genes predicted by at least three prediction tools were considered as potential targets of a specific miRNA. Consequently, the expression trends of 8 differentially expressed miRNAs (miR-128, miR-1271, miR-181a, let-7i, let-7c and let-7a) in SE animal group were found to show opposite expression pattern relative to their potential target genes (Table 3). Likewise, in the CE animals, one upregulated and eight downregulated miRNAs exhibited a reciprocal expression pattern with expression pattern of their corresponding potential target gene (Table 4). Similarly, both in SE and CE animals, the expression profile of 6 dysregulated miRNAs exhibited a reciprocal expression patterns with the expression patterns of their potential target genes (Table 5).

**Table 3:** MiRNAs downregulated (↓) only in SE animals and the expression patterns of their potential target genes

miRNAs	Potential target genes	The expression levels of target genes (in fold change) in the SE animals
miR-128↓	*PTHLH*	13.32
	*SLC7A5*	2.3
	*TGIF1*	2.2
	*AGTR1*	2.03
	*LBH*	1.99
	*TXNDC5*	1.89
miR-1271↓	*SLC7A5*	2.3
	*TGIF1*	2.2
	*PLOD2*	1.85
	*TIMP2*	-2.3
miR-1266↑	*GM2A*	-5
	*CYP39A1*	-2.6
	*CD68*	-1.4
	*CXCR4*	-2.6
	*CHRDL1*	-3
miR-1183↑	*RGS1*	-1.6
	*TIMP2*	-2.3
	*CXCR4*	-2.6
	*GM2A*	-5
miR-181a↓	*PTHLH*	13.32
	*SNAI2*	2.6
	*CKAP4*	2.5
	*SLC7A5*	2.3
	*COL6A3*	2.2
	*AGTR1*	2.03
	*TXNDC5*	1.89
let-7i↓	*SERF1A*	3.9
	*NPL*	2.5
	*CKAP4*	2.5
	*SLC7A5*	2.3
	*COL6A3*	2.2
let-7c↓	*LBH*	1.99
let-7a↓	*SERF1A*	3.9

Positive and negative values indicate up or down regulation of genes, respectively in SE compared to HE

**Table 4:** Down (↓) or upregulated (↑) miRNAs only in CE animals and the expression patterns of their target genes

miRNAs	Potential target genes	The expression levels (in fold change) of target genes in the CE animals
miR-592↓	*PTHLH*	21.3
	*SNAI2*	3.0
	*P2RY14*	7.0
	*CKAP4*	2.8
	*SLC7A5*	3.8
	*TGIF1*	2.3
	*LBH*	2.4
	*PLOD2*	2.3
	*DAPP1*	19.6
miR-608↑	*TGM2*	-2.6
	*TIMP2*	-2.6
miR-498↓	*INHBA*	19.3
	*PTHLH*	21.3
	*PCOLCE*	3.4
	*P2RY14*	7.0
	*CKAP4*	2.8
	*SLC7A5*	3.8
	*COL6A3*	2.5
	*LBH*	2.4
	*DAPP1*	19.6
	*RIMS1*	2.08
miR-449b↓	*CKAP4*	2.8
	*TXNDC5*	2.0
	*MAGED1*	2.3
miR-421↓	*COL6A3*	2.5
miR-30c↓	*SNAI2*	3.0
miR-222↓	*PTHLH*	21.3
	*LBH*	2.4
	*TXNDC5*	2.0
	*PLOD2*	2.3
miR-21↓	*PCOLCE2*	3.4
	*P2RY14*	7.0
	*REXO2*	*2.3*
	*AGTR1*	15.0
	*TXNDC5*	2.0

Positive and negative values indicate up and down regulation of genes, respectively in CE compared to HE animals

**Table 5:** MiRNAs downregulated (↓) both in CE and SE animals and the expression patterns of their potential target genes

miRNAs	Target genes	The expression levels of target genes (in fold change) in the CE animals	The expression levels of target genes (in fold change) in the SE animals
miR-196b↓	*COL1A1*	2.6	2.3
miR-151-5p↓	*TMEM45A*	2.7	2.7
	*SLC7A5*	3.8	2.3
	*LBH*	2.4	1.91
	*COL6A3*	2.5	2.2
	*MAGED1*	2.3	1.8
miR-1204↓	*TGFB1I1*	2.7	2.9
	*MLLT11*	2.51	2.7
	*SLC7A5*	3.8	2.3
miR-1203↓	*SLC7A5*	3.8	2.3
let-7f↓	*COL6A3*	2.5	2.2
	*LBH*	2.4	2.0
	*PTHLH*	21.3	13.3
	*SERF1A*	3.6	3.9
	*NPL*	4.0	2.5
	*CKAP4*	2.8	2.5
	*SLC7A5*	3.8	2.3
	*COL6A3*	2.5	2.2
	*LBH*	2.4	1.99
	*TXNDC5*	2.0	1.89
let-7e↓	*SERF1A*	3.6	3.9
	*TGFB1I1*	2.7	2.9
	*NPL*	4.04	2.5
	*CKAP4*	2.8	2.5
	*SLC7A5*	3.8	2.3
	*COL6A3*	2.5	2.2
	*LBH*	2.4	1.99
	*TXNDC5*	2.0	1.89

## Discussion

Postpartum uterine inflammation due to bacterial infection is one of the major problems impairing the productive and reproductive performance of dairy cows. Nevertheless, during bacterial infection, the endometrium may respond to the invasion of foreign body by activating or repressing certain biochemical and molecular signals. Therefore, understanding these molecular events could be a step forward to craft a potential roadmap for identifying diagnostic molecular markers that could be used as indicators of subclinical and/or clinical endometritis incidence. Previously, we have demonstrated the presence of limited endometrial gene expression alterations in dairy cows categorized as subclinical and healthy based on the threshold PMN value of > 0 or =0 [12]. Unlike to that study, however, in the current study, the effects of endometritis on the expression patterns of endometrial coding genes and small non coding genes were investigated using endometrial samples obtained from cows classified as subclinical (SE) or clinical endometritis (CE) based on clinical examination of the animals and histological evaluation of the corresponding endometrial samples. Based on this analysis, in the present study, about 29.0 % of cows were found to be affected by endometritis at day 52–70 postpartum which is lower compared to our previous report [12] and others [13, 14]. These differences might be associated with postpartum periods at which the cows were investigated and the methodological approaches employed to categorize cows. For instance, counting the proportion of polymorphonuclear neutrophils (PMNs) in endometrial cytology samples collected either by cytobrush or punch forceps has been the most commonly used diagnostic tool to identify cows affected by subclinical endometritis [13, 15–17]. However, classification of cows as healthy and subclinical endometritis depending on the proportion of endometrial PMNs seems to be inconsistent [12, 17–20] due to absence of consensus on the levels of PMN threshold. Therefore, in this study, we have employed both clinical diagnosis of the cow and histological evaluation of the endometrial biopsy to investigate the transcriptome and miRNome profile changes occurred in the uterine environment during endometritis infection. We used the GeneChip® Bovine Genome Array (Affymetrix, CA, USA) to unravel the alterations of the endometrial transcriptome profile in cows affected by subclinical or clinical endometritis. Subsequently, the expression levels of 203 and 28 gene transcripts were significantly altered in animals affected by clinical and subclinical endometritis. In addition, all most all gene transcripts that were significantly dysregulated in subclinical animals including *TGIF1, TGFB1I1, PTHLH, INHBA* and *MAOB*, CXCR4, *SLC16A11* and *NFIB* were also significantly dysregulated in cows affected by clinical endometritis. Nevertheless, 87 % differentially expressed genes in CE animals were not significantly altered in SE animal group. This may suggest that a presence of direct association between the level of uterine inflammation and the mRNA expression alterations within the uterine molecular milieu.

Since the microarray and qRT-PCR analysis were conducted on endometrial biopsies, it was unclear whether the gene expression alterations specific to endometrial epithelial or stroma cells or in both of the cell types. Therefore, to verify this and to further validate the array and the qRT-PCR data, we have measured the expression level of randomly selected candidate genes whose expressions were altered in clinical and/or subclinical animal groups in endometrial epithelial and stroma cells challenged with subclinical or clinical endometritis equivalents doses of LPS. For instance, the expression levels of MLLT11, *INHBA*, and *PTHLH* were significantly upregulated in both clinical and subclinical endometritis animal groups and the expression levels of these genes were also found to be increased in endometrial epithelial and stroma cells challenged with clinical or subclinical equivalent LPS doses. Interestingly, the mRNA increment seems to be more pronounced in epithelial than stroma cells. On the other hand, the genes, namely *JUN*, *PTGDS,* and *EMID2* whose expression was significantly reduced in cows affected by clinical endometritis, was also decreased in epithelial cells challenged with SE and CE equivalent doses of LPS suggesting that alteration of these genes in endometrium were caused by inflammation.

Parallel to the global mRNA expression analysis, we have analyzed the endometrial miRNA expression profile changes induced by clinical or subclinical endometritis. In fact miRNAs are believed to regulate the gene expression either by degrading the mRNA or inhibiting the protein translation. Furthermore, the miRNAs are implicated in various biological processes and believed to be used as a diagnostic markers of preeclampsia [21] and multiple forms of cancer [22–26]. In line to this, in the current study, we analyzed the miRNA expression patterns in animals affected by subclinical or clinical endometrium using miRCURY LNA^TM^ Universal RT miRNA. Although, miRCURY LNA^TM^ Universal RT miRNA array consisted of human miRNAs, we detected a wide range of miRNAs in bovine endometrial samples due to the conserved nature of miRNAs between several species [27]. Previously, we have also reported a high detection rate of circulatory miRNAs in bovine follicular fluids using the same PCR array platform [28]. However, in the current study, our focus was indeed intended to identify endometrial miRNAs which are either repressed or activated during subclinical and/or clinical endometrial incidences. In line to this, we have confirmed that although the dysregulation of miRNA expression patterns both in SE and CE seem to have a similar pattern, significant analysis revealed altered expression profiles of 102 miRNAs including the let-7 family (let-7a, let-7c, let-7d, let-7d*, let-7e, let-7f and let-7i) in SE group 35 miRNAs including let-7e, let-7f, miR1265 and miR-608 in CE animals. Moreover, 90 and 80 % of the affected miRNAs were downregulated in subclinical and clinical endometritis animal groups, respectively. Other report [29] also showed that bovine mammary epithelial cells challenged with *Staphylococcus aureus* (*S. aureus*) or *Escherichia coli (E. coli)* resulted in dysregulation of 17 miRNAs of which five miRNAs including miR-148, miR-486 and let-7a-5p were unique to E. coli while four miRNAs including miR-23a and miR-99b were unique to S. aureus. Similarly, in the current study, miR-148b, miR-486-5p, miR-23b, miR-99b and members of the let-7 families were altered in animals affected by subclinical endometritis. Moreover, other miRNAs dysregulated in the current study, namely miRNA 128, let-7e, let-7d, miRNA and miRNA- 652 were also found to be altered in bovine mammary epithelial cell cultured in the presence of *streptococcus uberis* [30]. Thus, some of the observed alterations in the expression of distinct miRNAs in animals affected by subclinical or clinical endometritis could be due to bacterial infections, such as E*. coli*, streptococcus uberis and S. aureus. Nevertheless, the question is to what extent the differentially expressed miRNAs could be associated with differentially expressed genes in animals affected by subclinical or clinical endometritis. Thus, to answer this question and to get an overview about this, we have performed in silico target prediction analysis. Accordingly, the result evidenced that the miRNAs including the let-7 family members showed inverse expression profiles with the expression patterns of their potential target genes. For instance, the expression level of let-7f miRNA was downregulated both in subclinical and clinical animal groups and the expression patterns of its target genes, namely SERF1A and TGFB1I1 were upregulated in both animal groups compared to healthy animals. In deed, one of the interesting findings of the current study was the downregulation of the let-7 miRNA family in animals affected by endometritis. The let-7 miRNA family is believed to be involved in a wide range of cellular functions and is implicated in modulation of several diseases. These miRNAs were first discovered in *Caenorhabditis elegans* and their function is believed be conserved across species and maintaining the normal expression patterns of these miRNAs could be a potential option in cancer therapeutics [31, 32]. Altered expression of let-7 might result in abnormal cell development and cancer [33]. For instance, overexpression of let-7a was found to inhibit tumour development [34] and inhibition may increase chemotherapy induced apoptosis [35]. Similarly, overexpression of let-7b may reduce cell proliferation and G2/M phase arrest [34] and increased level of let-7b and let-7c using miRNA mimics helped the human hepatocytes to resist against oxidant injury induced by tert-butyl-hydroperoxide [36]. Furthermore, members of the Let-7 miRNA family are believed to regulate the expression of cytokines which are directly or indirectly involved in host-functions [37–41]. Therefore, expression alterations in the Let-7 family miRNAs in cows suffering from endometritis may demonstrate the consequences of bacterial infections on the expression pattern of these miRNAs. However, the functional role of *let-7* family miRNAs with respect to immune responses of the bovine endometrium due to bacterial inflammation needs further investigation.

## Conclusion

In conclusion, clinical and/or subclinical endometritis induce alterations in the expression level of bovine endometrial mRNA transcripts that are associated with the immune system, cell adhesion, regulation of apoptotic signaling pathway, G-protein coupled receptor signaling pathway and chemotaxis. Moreover, subclinical and clinical endometritis also altered the expression pattern of several endometrial miRNAs in postpartum cow. All in all, the results of this study unraveled the effect of subclinical and clinical endometritis on the endometrial transcriptome and miRNome profile and associated molecular pathways. These molecular dysregulations in turn may disturb the homeostasis of the uterine environment as well as uterine receptivity.

## Methods

### Animals and samples

Forty five lactating Holstein Friesian cows at 42–60 days postpartum were used for the study. Cows were housed in a free-stall barn with slotted floors and cubicles, lined with rubber mats and received a total mixed ration. The experimental animals have no previous records of mastitis and they didn’t have a record of retained placenta. Moreover, the animals have normal ovarian activity and they all have similar parity. Handling and management of experimental animal was performed according to the rules and regulations of the German law of animal protection. Moreover, the experiment was approved by the Animal Welfare Committee of the University of Bonn with proposition number 84-02.05.20.12.075. The ovaries of the cows used for the experiment were examined by ultrasonography at 42–60 days postpartum. Animals with the presence of a corpus luteum or a dominant follicle (diameter ≥ 9 mm), received one dose of GnRH (2.5 ml Receptal®) followed by PGF2α (2.0 ml Estrumate®) seven days later to induce estrous. Following this, 3 days later (day 52–70), cows were examined by an experienced veterinarian for signs of clinical endometritis. Briefly, all cows were examined by rectal palpation to grade the size of the uterus on a 3-point scale (I, II, III) with grade III uteri representing the bigger ones. Likewise, the size of the left (L) and the right (R) uterine horn were compared to assess the symmetry of the uterus. To that regard, the uterus was classified in to 3 conditions, namely the left horn bigger than the right one (L > R), both horns being of equal size (L = R) and the left horn smaller than the right one (L < R). In addition, the uterine cavity was checked for any signs of fluid discharge. Moreover, all cows were subjected to vaginoscopic inspection to evaluate the color of the vaginal mucosa (pinkish vs. hyperaemic), grade of wetness (on a 5-point scale (I-V) with grade 5 vagina representing the wettest ones), size of the external opening of the cervix and the presence of vaginal discharge. Collectively, all these indications were summarized as a clinical diagnosis with respect to endometrial health status of the cow. The endometrial biopsy samples were then collected using a uterine biopsy punch forceps. The biopsies collected from each cow were bisected and a part was placed in RNA later for further molecular analysis, the remaining part was used for histological analysis. Moreover, the biopsies were analyzed for presence of fibrosis and presence of immune cells before categorizing the corresponding endometrium samples as healthy, clinical or subclinical endometritis.

### Histological evaluation of the endometrial biopsies

**Fig. 11:**
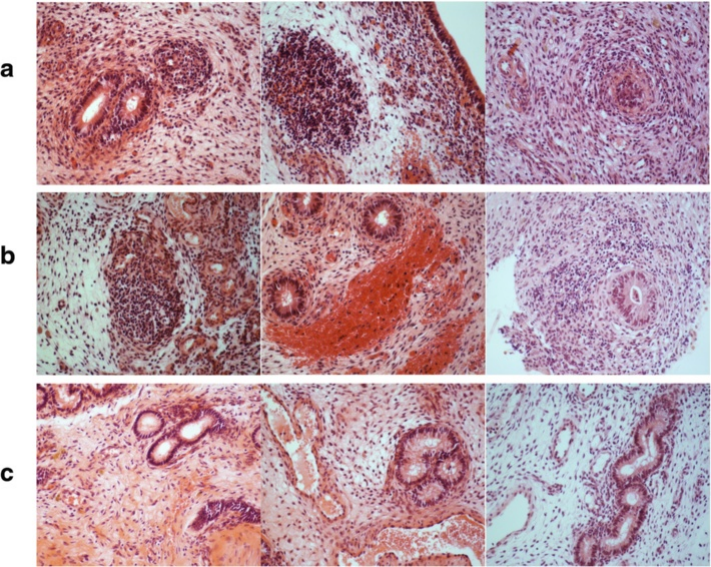
Histological sections of endometrial samples of which the corresponding endometrial biopsies which were used for global mRNA and miRNA expression analysis from cows classified as suffering from clinical endometritis (**a**), subclinical endometritis (**b**) and healthy animals (**c**)

To categorize the experimental animals as healthy, clinical or subclinical endometritis group, the corresponding endometrial biopsy samples were fixed in 4 % formalin for 24 h and then rinsed in PBS before being embedded in paraffin in an automated system. From the resulting paraffin blocks, 3 μm microtome sections were produced and deparaffinized in xylol. Then the sections were hematoxylin-eosin stained according to routine methods and examined under a light microscope by one observer (CP). After inspection of the total area of each section and qualitative evaluation, the animals were assigned to the following diagnoses: in apparent or inflammation (acute inflammation, or chronic inflammation). The criteria were 1) the presence, amount and type of leukocytes, 2) the occurrence of solitary lymph follicles, 3) the amount of endo- and extravascular erythrocytes, 4) the degree of glandular fibrosis and luminal fibrin deposition. Acutely inflamed endometrium was characterized by a strong infiltration with leukocytes (majority neutrophil granulocytes) and erythrocytes as well as the presence of fibrin depositions in the uterine lumen. A chronic affection was diagnosed when mainly lymphocytes were found, solitary lymph follicles were present and endometrial glands showed signs of fibrosis. In contrast, endometrium diagnosed as healthy contained only very few leukocytes and no solitary lymph follicles. The histological sections of endometrial samples of cows of which the corresponding endometrial samples were used for global mRNA and miRNA expression analysis are shown in Fig. 11.

### Classification of the endometrial biopsy samples

The endometrial biopsy samples were classified as healthy (HE), subclinical endometritis (SE) and clinical endometritis (CE) based on the results of veterinary clinical examination of the cows and the histological analysis of the corresponding endometrial biopsies. Thus, endometrial biopsies derived from cows that did not show signs of clinical endometritis and no indications of inflammation in their corresponding endometrial biopsies were classified as HE groups and those biopsies obtained from cows which were clinically healthy, but showed signs of inflammation based on histological evaluation of the biopsies were considered as the SE group and endometrial samples derived from cows that exhibited evidence of endometritis both in clinical assessment and histological evaluation were classified as CE group (Table 6).

**Table 6:** Classification of experimental animals as HE, SE or CE based on clinical examination of the animals and histological evaluation of the corresponding endometrial biopsies

Animal Nr.	Rectal examination				Vaginoscopic examination						
	Size (G I-III)	Symmetry (L vs. R)	Contractility (I-III)	Uterine charge (yes/no)	Mucosal color	Wetness (I-V)	Cervix opening (mm)	Discharge (yes/no)	Clinical examination	Histological classification	Overall classification
245	III	L > R	I	no	pinkish	II	3	no	healthy	in apparent	HE
534	II	L = R	I	no	pinkish	III	0	no	healthy	in apparent	HE
549	IV	L = R	I-II	no	hyperaemic	IV	6	no	healthy	in apparent	HE
665	II	L < < R	II	no	pale	III	6	no	healthy	in apparent	HE
328	III	L > R	I	no	pinkish	III	6	no	healthy	in apparent	HE
613	II	L = R	II	no	pinkish	III	3	no	healthy	in apparent	HE
627	II	L = R	I	no	pinkish	III	0	no	healthy	chronic inflammation	SE
602	II	L > R	I I	?	pinkish	II	3	no	healthy	in apparent	HE
603	II	L = R	I	no	pinkish	II	0	no	healthy	acute inflammation	SE
550	III	L < R	II	yes	pinkish	III	3	yes	sick	chronic inflammation	CE
369	II-III	L > R	I-II	yes	pinkish	II	6	no	healthy	chronic inflammation	SE
444	II-III	L > R	K II	no	pinkish	III	0	no	healthy	in apparent	HE
459	II	L = R	I-II	no	pinkish	II	0	no	healthy	in apparent	HE
506	III	L < < R	?	no	pinkish	III	3	no	healthy	in apparent	HE
432	III	L > R	II-III	no	pinkish	II	3	no	healthy	in apparent	HE
470	II	L = R	I	no	pinkish	III	3	no	healthy	in apparent	HE
445	III	L < R	II	no	pinkish	III	6	no	healthy	in apparent	HE
443	?	L < R	I-II	no	pinkish	II	3	no	healthy	?	?
347	II-III	L = R	II	no	pinkish	III	6	no	healthy	in apparent	HE
329	IV	L < R	I-II	yes	pinkish	III	3	yes	sick	chronic inflammation	CE
755	I-II	L = R	II	no	hyperaemic	II	3	yes	sick	chronic inflammation	CE
702	I	L = R	II	no	pinkish	II	0	no	healthy	in apparent	HE
639	I	L = R	I-II	no	pinkish	III	0	no	healthy	inflammation	SE
618	I	L > R	II	no	pinkish	II	0	no	healthy	inflammation	SE
749	I-II	L = R	II	no	hyperaemic	III	4.5	yes	sick		HE
721	I-II	L = R	II	yes	hyperaemic	IV	9	no	healthy	inflammation	SE
450	I-II	L = R	II-III	no	pinkish	II	0	no	healthy	in apparent	HE
469	I	L = R	III	no	pinkish	III-IV	7.5	no	healthy	in apparent	HE
723	I-II	L = R	II-III	no	pinkish	III-IV	6	no	healthy	in apparent	HE
737	I	L = R	I-II	yes	pinkish	IV	9	yes	sick	in apparent	HE
642	II	L = R	II	no	pinkish	II-III	6	yes	sick	acute inflammation	CE
665	III	L > R	II	no	pinkish	III	3	no	healthy	in apparent	HE
615	II-III	L = R	I-II	no	pinkish	III	3	no	healthy	in apparent	HE
764	I	L = R	I-II	no	pinkish	II	3	no	healthy	in apparent	HE
725	I-II	L = R	II	no	pinkish/hyperaemic	IV	7.5	no	healthy	?	?
740	II	L > R	II	no	blassrosa	II	6	no	healthy	?	?
606	I-II	L = R	II	no	pinkish	III	7.5	yes	sick	?	?
522	III	L < < R	II	no	pinkish	II	3	no	healthy	in apparent	HE
727	I-II	?	I-II	no	pinkish	II	0	no	healthy	in apparent	HE
729	I-II	L = R	I-II	no	pinkish	II	0	no	healthy	in apparent	HE
728	II	L = R	I-II	no	pinkish	II	0	no	healthy	?	?
753	II	L = R	II-III	no	pinkish	III-IV	3	no	healthy	in apparent	HE
767	II	L < R	I-II	no	pale	I	0	no	healthy	?	?
736	I	L = R	II	no	pinkish	II	3	yes	sick	?	?
696	II-III	l < R	I-II	yes	hyperaemic	IV	9	yes	sick	acute inflammation	CE

*L > R* left horn larger than right uterine horn, *L < R* right horn larger than left uterine horn

### RNA isolation from endometrial samples

Total RNA enriched with miRNA was isolated from three biological replicates of SE, CE or HE cows using miRNeasy mini kit (QIAGEN GmbH, Hilden, Germany). Each cow from each animal group was considered as one biological replicate. The concentration of the RNA was analyzed using the Nanodrop 8000 Spectrophotometer (Thermo Fisher Scientific Inc, DE, USA). The RNA quality was evaluated using Agilent 2100 Bioanalyzer with RNA 6000 Nano LabChip® Kit (Agilent Technologies Inc, CA, USA).

### Array hybridization and scanning

The total RNA samples isolated from each SE, CE and HE in three biological replicates were subjected to gene expression analysis using the GeneChip bovine Genome array (Affymetrix, CA, USA). For this, we performed RNA amplification, cDNA synthesis, labelling and array hybridization for each of the three cows of SE, CE or HE group according to the recommendations and suggestion of the GeneChip®3′ IVT Express Kit (Affymetrix, CA, USA). Three hybridizations were preformed for each SE, CE or HE animals and the three hybridization represented the biological replicates correspond to three animals of SE, CE or HE group. The array slides were then washed and stained using the Fluidics Station 450/250 (Affymetrix, CA, USA) following the GeneChip® expression wash, stain and scan user manual. After 16 h of hybridization, the arrays were scanned with the GeneChip™3000 laser confocal slide scanner (Affymetrix, CA, USA) integrated with GeneChip® Operating System (GCOS). The signal intensity of the control probes were monitored during array scanning

### Array data analysis and visualization

After scanning, the data was normalized using GC robust multi-array average analysis (GCRMA) [42] using bioconductor packages (http://bioconductor.org) in the R environment (www.r-project.org). Linear models for microarray data (LIMMA) [43] was employed to identify differentially expressed genes in SE and CE relative to the HE animals Benjamini–Hochberg procedure of false discovery rate *p* value adjustment was used during analysis [44]. The G- profiler analysis tool was used to investigate the functional annotation of differentially expressed genes [45]. The Heatmaps and clustering of differentially expressed genes were constructed using PermutMatrix software [46].

### Quantitative Reverse Transcription Real Time polymerase Chain Reaction (qRT-PCR)

The expression profiles of randomly selected differentially expressed genes were validated using SYBR Green based qRT-PCR using sequence specific primers (Table 7) designed using the online primer design tool (http://frodo.wi.mit.edu/primer3/). The specificity and identity of the gene fragments amplified by each primer pair were verified by sequencing their PCR products using GenomeLab™ GeXP Genetic Analysis System (Beckman Coulter). Afterwards, the cDNA samples from SE, CE and HE sample group were synthesized by reverse transcription of equal amount of total RNA from each endometrial biopsy. The qRT-PCR was then performed in 20 μl reaction volume containing iTaq SYBR Green Supermix with ROX (Bio-Rad laboratories, Munich, Germany), the cDNA samples, the specific forward and reverse primer in the StepOnePlus™ Real-Time PCR Systems (Applied Biosystems, Foster city, CA). At the end of each PCR reaction, the specificity the amplification was confirmed by evaluating the dissociation curve. The abundance of each transcript in each sample was determined using a comparative threshold cycle comparative Ct (2^−ΔΔCT^) method as described previously [47]. The data obtained from qRT-PCR was analyzed after the Ct value of the target genes was normalized with the Ct value of Glyceraldehyde-3-phosphate dehydrogenase (*GAPDH*). The qRT-PCR was performed in three biological and two technical replicates. The Student’s *t*-test or the least significant difference test procedure was employed to detect the mRNA expression differences between the samples. Differences with *p* < 0.05 were considered as significant.

**Table 7:** Genes and corresponding primers used for validation of the gene expression data using qRT-PCR

Accession No.	Gene symbol	Gene title	Primer 5‘–3‘	Bp
NM_001077009	*P2RY14*	Bos taurus purinergic receptor P2Y, G-protein coupled, 14	F TATGCCAGCCATTTAGAGAGG	137
			R GGAGGTGGGAATTCACAGAG	
NM_174363.2	*INHBA*	Bos taurus inhibin, beta A	F GCAAGGTCAACATCTGCTGTA	
			R TACAACATGGACATGGGTCTC	
NM_177944	*MAOB*	Bos taurus monoamine oxidase B	F CTATGGCTTTGTGCTTGTCCT	253
			R TCCTGAGAGATGGGATAAAGC	
NM_001024563.1	*KCNB2*	Bos taurus potassium voltage-gated channel, Shab-related subfamily, member 2	F CTCTTTACTTTCTCCGCCAGA	282
			R CATCTTGCACCCTTCTTGACT	
NM_001083770.1	*C16H1orf95*	Bos taurus chromosome 16 open reading frame, human C1orf95	F GATAGACAGATTCCTGCCTGGT	268
			R TGTTGAGTGTAATGGGGAAGG	
NM_001077858.2	*CTPS*	Bos taurus CTP synthase	F AGGAAGAGGGAAACCAGAGAC	277
			R CCCTTGAGCAAAGCTGTCTAC	
NM_174753.1	*PTHLH*	Bos taurus parathyroid hormone-like hormone	F AGCAGAGACCTTCAGAAGACG	267
			R GAAATTCAGCAGCACCAAGA	
NM_001075835.1	*NKIRAS1*	Bos taurus NFKB inhibitor interacting Ras-like 1	F GACCTTTCGGAACAGAGACAG	265
			R CACGGGTACCTAGAGGCAGT	
NM_001101105.2	*TRIB1*	Bos taurus tribbles homolog 1 (Drosophila)	F CGTGTATACCTCACGCACTGT	287
			R CAGCAAACCCAGAGTCCTTAG	
AF399642.1	*CXCR4*	Bos taurus CXC chemokine receptor 4, complete cds	F CCACTCCAAAGGCTATCAGAA	287
			R CTCTGCTCACAGAGGTGAGTG	
NM_173879.2	*F5*	Bos taurus coagulation factor V (proaccelerin, labile factor)	F GAACGGACTGGAAACCTTACA	252
			R GCCCACTCTAAGTGGTTTGAT	
NM_174791.4	*PTGDS*	Bos taurus prostaglandin D2 synthase 21 kDa (brain)	F AGGAAAGACCAGTGTGAGACG	285
			R GAACACAATGCCTTCCTCTGT	
XM_592894.6	*CHRDL1*	PREDICTED: Bos taurus chordin-like 1	F CCAGGTGTTCTCTGAAAGCTC	319
			R GGTACTTATGGGCTTTGCTTG	

*BP* the lengths of the DNA sequence (in base pair)

### MiRNA expression pattern analysis in SE, CE and HE animals

The expression of patterns of miRNAs in SE, CE and HE animals were investigated using the Exiqon microRNA PCR Human Panels (I + II) array technology. The cDNA was synthesized from 88 ng of total RNA from each sample following the manufacturer’s protocol. Before performing qRT-PCR reaction, the cDNA samples were diluted 100-fold and mixed with ready to use SYBR-Green mix. Then the master mix was robotically distributed on 384 well PCR plate containing miRNA specific primers. The real time qRT-PCR was run in a 7900H thermo cycler (ABI) using the following thermal-cycling parameters: 95 °C for 10 min, 40 cycle of 95 °C for 10 s, 60 °C for 1 min followed by a melting curve analysis. The PCR data was analyzed using web-based PCR array data analysis software RT^2^ profile PCR array data analysis (http://pcrdataanalysis.sabiosciences.com/pcr/arrayanalysis.php) and normalization was performed using the geometric mean of miR-23a, miR-103, miR-191 and SNORD49A. To minimize the potential noise, miRNAs with Ct value higher than 36 were considered as undetected. The data was generated from three biological replicates of the SE, CE and HE sample groups and the three biological replicates correspond to the samples derived from three animals of SE, CE or HE group. Following this, comparative analysis of differentially expressed miRANs and differentially expressed genes analyzed using miRecords (http://c1.accurascience.com/miRecords/), an online animal miRNA-target interaction tool which integrates 12 miRNA-target prediction tools, including DIANA-microT, MicroInspector, miRanda, MirTarget2, miTarget, PicTar, PITA, and TargetScan.

### Experssion analysis of candidate mRNA transcripts and miRNAs in endometrial epithelial and stroma cells challenged with lipopolysacharide (LPS) in vitro

Endometrial cell culture was preformed to validate selected candidate genes and miRNAs obtained from the array results. For this, endometrial cell were collected from healthy non pregnant cows with no evidence of genital disease or microbial infection. The endometrial samples were cut into pieces and incubated in 25 ml sterile digestive solution [50 mg trypsin III (Gibco)], 50 mg collagenase II (Sigma), and 10 μl deoxyribonuclease I (Qiagen) in 100 ml phosphate buffer saline at 37 °C. The cell suspension was then filtered in a 40-μm mesh. The DMEM/F-12 medium supplemented with 10 % fetal bovine serum was added to the filtrate and centrifuged at 100 × *g* for 10 min. After two consecutive washes, the cells were suspended in DMEM/F-12 containing 10 % fetal bovine serum, 10 μl/ml penicillin streptomycin and 10 μl/ml fungizol and plated at a density of 1 × 10^5^ cells/ml in 24-well plates. The cells were then cultured at 37 °C, 5 % CO_2_ in a humidified incubator.

### Stromal and epithelial cell separation and *lipopolysaccharide (LPS)* challenge

The endometrial stromal and epithelial cells were separated after 18 h of culture. For this, the cell suspension was collected leaving the attached cells in the plate. Those cells attached to the plate after 18 h of culture were considered to be the stromal cells [48], while others which were in the cell suspension were considered as epithelial cells. The cell suspension was then plated to the new 24 well plate and cultured at 37 °C, 5 % CO2 in a humidified incubator to allow the epithelial cells to adhere [49]. In addition, the stromal and epithelial cell identity was monitored based on their morphology [48]. At the stage of confluence, the epithelial and stromal cells were challenged with clinical (3 μg/ml) or subclinical (0.5 μg/ml) doses of ultra pure LPS from E. coli 0111:B4 strain- TLR4 ligand (Invitrogen) for 24 h. These doses were similar to the uterine lumen of subclinical and clinical endometritis affected animals [50]. The supernatants and harvested cells were kept in –80 °C until further use. The cell culture supernatant was used for measuring prostaglandin E_2_ (PGE_2_) and prostaglandin F_2_ alpha (PGF_2α_) levels using Enzyme-linked Immunosorbent Assay (ELISA). The absence of immune cells in the primary epithelial cell and stromal cultures was confirmed by measuring the expression level of CD45 which is pan-leukocyte marker [51]. Afterwards, the expression levels the candidate differentially expressed gene transcripts and miRNAs were quantified in both endometrial and epithelial cells challenged with SE or CE equivalent doses of LPS.

### Availability of supporting data

The raw and normalized data gene expression data used for this manuscript have been deposited in the Gene Expression Omnibus repository, with GEO accession number GSE74987 (http://www.ncbi.nlm.nih.gov/geo/query/acc.cgi?acc=GSE74987).

## Additional files

Additional file 1: Table S1. The list of differentially expressed genes between CE and HE animals along with their fold change, *p* values and FDR. (XLSX 31 kb) Additional file 2: Table S2. The list of differentially expressed genes between SE and HE animals along with their fold change, *p* values and FDR. (XLSX 12 kb) Additional file 3: Figure S1. The expression pattern and the hierarchical clustering of differentially expressed miRNAs between SE and HE animal groups. (TIF 130 kb) Additional file 4: Table S3. The list of differentially expressed miRNAs between SE and HE animal groups. (XLSX 12 kb)

## Abbreviations

CE: clinical endometritis
FDR: false discovery rate
HE: healthy animals
LPS: lipopolysaccharide
miRNA: microRNA
SE: subclinical endometritis

## References

[1] Fenwick MA, Fitzpatrick R, Kenny DA, Diskin MG, Patton J, Murphy JJ, Wathes DC (2008). Interrelationships between negative energy balance (NEB) and IGF regulation in liver of lactating dairy cows. Domest Anim Endocrinol.

[2] Wathes D, Fenwick M, Cheng Z, Bourne N, Llewellyn S, Morris D, Kenny D, Murphy J, Fitzpatrick R (2007). Influence of negative energy balance on cyclicity and fertility in the high producing dairy cow. Theriogenology.

[3] Toni F, Vincenti L, Ricci A, Schukken YH (2015). Postpartum uterine diseases and their impacts on conception and days open in dairy herds in Italy. Theriogenology.

[4] Sordillo LM, Contreras GA, Aitken SL (2009). Metabolic factors affecting the inflammatory response of periparturient dairy cows. Anim Health Res Rev.

[5] Cheong SH, Nydam DV, Galvao KN, Crosier BM, Gilbert RO (2011). Cow-level and herd-level risk factors for subclinical endometritis in lactating Holstein cows. J Dairy Sci.

[6] Thatcher W, Santos J, Silvestre F, Kim I, Staples C (2010). Perspective on physiological/endocrine and nutritional factors influencing fertility in post‐partum dairy cows. Reprod Domest Anim.

[7] Hammon DS, Evjen IM, Dhiman TR, Goff JP, Walters JL (2006). Neutrophil function and energy status in Holstein cows with uterine health disorders. Vet Immunol Immunopathol.

[8] Galvão K, Flaminio M, Brittin S, Sper R, Fraga M, Caixeta L, Ricci A, Guard C, Butler W, Gilbert R (2010). Association between uterine disease and indicators of neutrophil and systemic energy status in lactating Holstein cows. J Dairy Sci.

[9] Sheldon IM, Williams EJ, Miller AN, Nash DM, Herath S (2008). Uterine diseases in cattle after parturition. Vet J.

[10] Ghasemi F, Gonzalez-Cano P, Griebel P, Palmer C (2012). Proinflammatory cytokine gene expression in endometrial cytobrush samples harvested from cows with and without subclinical endometritis. Theriogenology.

[11] Gabler C, Drillich M, Fischer C, Holder C, Heuwieser W, Einspanier R (2009). Endometrial expression of selected transcripts involved in prostaglandin synthesis in cows with endometritis. Theriogenology.

[12] Hoelker M, Salilew-Wondim D, Drillich M, Christine G-B, Ghanem N, Goetze L, Tesfaye D, Schellander K, Heuwieser W (2012). Transcriptional response of the bovine endometrium and embryo to endometrial polymorphonuclear neutrophil infiltration as an indicator of subclinical inflammation of the uterine environment. Reprod Fert Dev.

[13] Gilbert RO, Shin ST, Guard CL, Erb HN, Frajblat M (2005). Prevalence of endometritis and its effects on reproductive performance of dairy cows. Theriogenology.

[14] Plöntzke J, Madoz L, De la Sota R, Drillich M, Heuwieser W (2010). Subclinical endometritis and its impact on reproductive performance in grazing dairy cattle in Argentina. Anim Reprod Sci.

[15] Kasimanickam R, Duffield T, Foster R, Gartley C, Leslie K, Walton J, Johnson W (2004). Endometrial cytology and ultrasonography for the detection of subclinical endometritis in postpartum dairy cows. Theriogenology.

[16] Sheldon IM, Lewis GS, LeBlanc S, Gilbert RO (2006). Defining postpartum uterine disease in cattle. Theriogenology.

[17] Barlund C, Carruthers T, Waldner C, Palmer C (2008). A comparison of diagnostic techniques for postpartum endometritis in dairy cattle. Theriogenology.

[18] Galvão K, Frajblat M, Brittin S, Butler W, Guard C, Gilbert R (2009). Effect of prostaglandin F2alpha on subclinical endometritis and fertility in dairy cows. J Dairy Sci.

[19] Kasimanickam R, Duffield T, Foster R, Gartley C, Leslie K, Walton J, Johnson W (2005). The effect of a single administration of cephapirin or cloprostenol on the reproductive performance of dairy cows with subclinical endometritis. Theriogenology.

[20] Drillich M, Tesfaye D, Rings F, Schellander K, Heuwieser W, Hoelker M (2012). Effects of polymorphonuclear neutrophile infiltration into the endometrial environment on embryonic development in superovulated cows. Theriogenology.

[21] Freeman DJ, Tham K, Brown EA, Rumley A, Lowe GD, Greer IA (2008). Fetal corticotrophin-releasing hormone mRNA, but not phosphatidylserine-exposing microparticles, in maternal plasma are associated with factor VII activity in pre-eclampsia. J Thromb Haemost.

[22] Kopreski MS, Benko FA, Kwak LW, Gocke CD (1999). Detection of tumor messenger RNA in the serum of patients with malignant melanoma. Clin Cancer Res.

[23] Taylor DD, Gercel-Taylor C (2008). MicroRNA signatures of tumor-derived exosomes as diagnostic biomarkers of ovarian cancer. Gynecol Oncol.

[24] Mitchell PS, Parkin RK, Kroh EM, Fritz BR, Wyman SK, Pogosova-Agadjanyan EL, Peterson A, Noteboom J, O’Briant KC, Allen A (2008). Circulating microRNAs as stable blood-based markers for cancer detection. Proc Natl Acad Sci U S A.

[25] Rabinowits G, Gercel-Taylor C, Day JM, Taylor DD, Kloecker GH (2009). Exosomal microRNA: a diagnostic marker for lung cancer. Clin Lung Cancer.

[26] Dasi F, Lledo S, Garcia-Granero E, Ripoll R, Marugan M, Tormo M, Garcia-Conde J, Alino SF (2001). Real-time quantification in plasma of human telomerase reverse transcriptase (hTERT) mRNA: a simple blood test to monitor disease in cancer patients. Lab Invest.

[27] Berezikov E (2011). Evolution of microRNA diversity and regulation in animals. Nat Rev Genet.

[28] Sohel MMH, Hoelker M, Noferesti SS, Salilew-Wondim D, Tholen E, Looft C, Rings F, Uddin MJ, Spencer TE, Schellander K (2013). Exosomal and Non-exosomal transport of extra-cellular microRNAs in follicular fluid: implications for bovine oocyte developmental competence. PLoS One.

[29] Jin W, Ibeagha-Awemu EM, Liang G, Beaudoin F, Zhao X (2014). Transcriptome microRNA profiling of bovine mammary epithelial cells challenged with Escherichia coli or Staphylococcus aureus bacteria reveals pathogen directed microRNA expression profiles. BMC Genomics.

[30] Lawless N, Foroushani AB, McCabe MS, O’Farrelly C, Lynn DJ (2013). Next generation sequencing reveals the expression of a unique miRNA profile in response to a Gram-positive bacterial infection. PLoS One.

[31] Boyerinas B, Park SM, Hau A, Murmann AE, Peter ME (2010). The role of let-7 in cell differentiation and cancer. Endocr Relat Cancer.

[32] Wang Y, Hu X, Greshock J, Shen L, Yang X, Shao Z, Liang S, Tanyi JL, Sood AK, Zhang L (2012). Genomic DNA copy-number alterations of the let-7 family in human cancers. PLoS One.

[33] Roush S, Slack FJ (2008). The let-7 family of microRNAs. Trends Cell Biol.

[34] Liu C, Kelnar K, Vlassov AV, Brown D, Wang J, Tang DG (2012). Distinct microRNA expression profiles in prostate cancer stem/progenitor cells and tumor-suppressive functions of let-7. Cancer Res.

[35] Meng F, Henson R, Wehbe-Janek H, Smith H, Ueno Y, Patel T (2007). The MicroRNA let-7a modulates interleukin-6-dependent STAT-3 survival signaling in malignant human cholangiocytes. J Biol Chem.

[36] Hou W, Tian Q, Steuerwald NM, Schrum LW, Bonkovsky HL (2012). The let-7 microRNA enhances heme oxygenase-1 by suppressing Bach1 and attenuates oxidant injury in human hepatocytes. Biochim Biophys Acta.

[37] Chen XM, Splinter PL, O’Hara SP, LaRusso NF (2007). A cellular micro-RNA, let-7i, regulates Toll-like receptor 4 expression and contributes to cholangiocyte immune responses against Cryptosporidium parvum infection. J Biol Chem.

[38] Sung SY, Liao CH, Wu HP, Hsiao WC, Wu IH, Yu J, Lin SH, Hsieh CL (2013). Loss of let-7 microRNA upregulates IL-6 in bone marrow-derived mesenchymal stem cells triggering a reactive stromal response to prostate cancer. PLoS One.

[39] Kumar M, Ahmad T, Sharma A, Mabalirajan U, Kulshreshtha A, Agrawal A, Ghosh B (2011). Let-7 microRNA-mediated regulation of IL-13 and allergic airway inflammation. J Allergy Clin Immunol.

[40] Iliopoulos D, Hirsch HA, Struhl K (2009). An epigenetic switch involving NF-kappaB, Lin28, Let-7 MicroRNA, and IL6 links inflammation to cell transformation. Cell.

[41] Swaminathan S, Suzuki K, Seddiki N, Kaplan W, Cowley MJ, Hood CL, Clancy JL, Murray DD, Mendez C, Gelgor L (2012). Differential regulation of the Let-7 family of microRNAs in CD4+ T cells alters IL-10 expression. J Immunol.

[42] Gharaibeh RZ, Fodor AA, Gibas CJ (2008). Background correction using dinucleotide affinities improves the performance of GCRMA. BMC Bioinformatics.

[43] Smyth GK, Gentleman R, Carey V, Dudoit S, Irizarry R, Huber W (2005). Limma: linear models for microarray data. Bioinformaticsand computational biology solutions using R and bioconductor.

[44] Benjamini Y, Hochberg Y (1995). Controlling the false discovery rate: a practical and powerful approach to multiple testing. Roy Statist Soc Ser:B.

[45] Reimand J, Kull M, Peterson H, Hansen J, Vilo J (2007). G:Profiler--a web-based toolset for functional profiling of gene lists from large-scale experiments. Nucleic Acids Res.

[46] Caraux G, Pinloche S (2005). PermutMatrix: a graphical environment to arrange gene expression profiles in optimal linear order. Bioinformatics.

[47] Livak KJ, Schmittgen TD (2001). Analysis of relative gene expression data using real-time quantitative PCR and the 2(-Delta Delta C(T)) Method. Methods.

[48] Fortier MA, Guilbault LA, Grasso F (1988). Specific properties of epithelial and stromal cells from the endometrium of cows. J Reprod Fertil.

[49] Kim JJ, Fortier MA (1995). Cell type specificity and protein kinase C dependency on the stimulation of prostaglandin E2 and prostaglandin F2 alpha production by oxytocin and platelet-activating factor in bovine endometrial cells. J Reprod Fertil.

[50] Herath S, Williams EJ, Lilly ST, Gilbert RO, Dobson H, Bryant CE, Sheldon IM (2007). Ovarian follicular cells have innate immune capabilities that modulate their endocrine function. Reproduction.

[51] Herath S, Fischer DP, Werling D, Williams EJ, Lilly ST, Dobson H, Bryant CE, Sheldon IM (2006). Expression and function of Toll-like receptor 4 in the endometrial cells of the uterus. Endocrinology.

