# Supplementary figures and images for: Clinical and subclinical endometritis induced alterations in bovine endometrial transcriptome and miRNome profile
Source: BMC Genomics. 2016 Mar 10;17:218. doi: 10.1186/s12864-016-2513-9 (PMC4785637; doi:10.1186/s12864-016-2513-9)

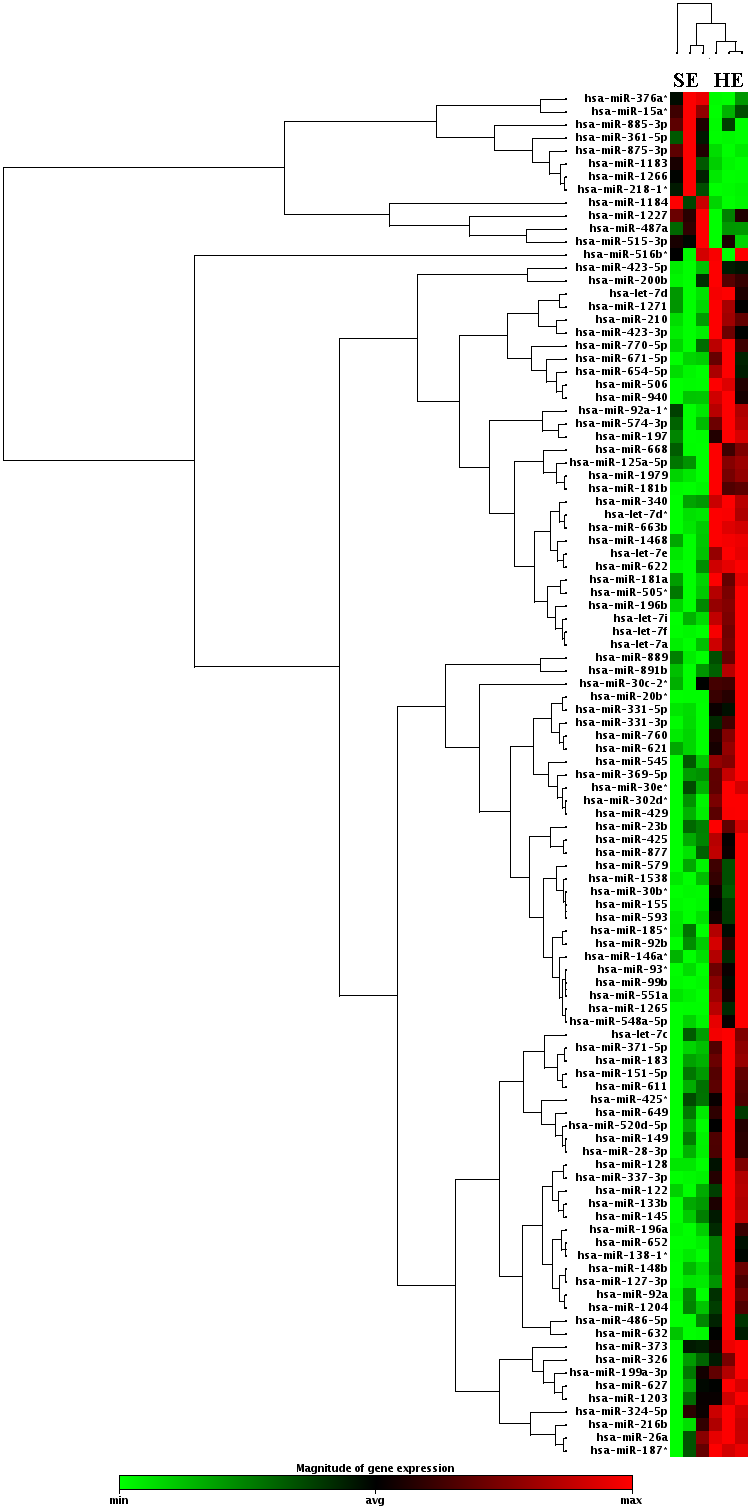

Supplement: Additional file 3: Figure S1. — The expression pattern and the hierarchical clustering of differentially expressed miRNAs between SE and HE animal groups. (TIF 130 kb) [file 12864_2016_2513_MOESM3_ESM.tif]
